# Governing antimicrobial resistance in Norwegian livestock farming to 2050: a participatory strategy development approach

**DOI:** 10.3389/fvets.2025.1616206

**Published:** 2025-08-14

**Authors:** Richard Helliwell, Lisa Boden, Eirik Magnus Fuglestad, Madelaine Norström, Anne Margrete Urdahl

**Affiliations:** ^1^Ruralis – Institute for Rural and Regional Research, Trondheim, Norway; ^2^Royal (Dick) School of Veterinary Studies, University of Edinburgh, Edinburgh, United Kingdom; ^3^Norwegian Veterinary Institute, Ås, Norway

**Keywords:** scenarios, futures, antimicrobial resistance, livestock farming, animal health, resilience, governance

## Abstract

Antimicrobial resistance (AMR) is a wicked problem with long-term and unpredictable impacts on human and animal health. Understanding how to govern AMR long-term, amidst evolving social, political, economic, technological and environmental changes which will impact livestock production, animal health and AMR risks is therefore critical. The study used scenario planning as a methodology for envisioning plausible future challenges and thus identify possible strategic responses. The national context for this research was Norway, a stable, high-income country which has achieved low antibiotic use and low AMR prevalence in livestock farming through nearly 30 years of concerted industry and state actions. Working with Norwegian agricultural, animal and public health stakeholders, the scenario approach was motivated by the question of how to maintain existing governance capabilities and outcomes in an uncertain future. This is the first scenario planning study to explore stakeholder perceptions about important change drivers and strategies to manage uncertainties for AMR governance in the Norwegian livestock industry. Participants identified three critical drivers of change (state resource prioritisation of agriculture, trust in institutions, global geopolitical conditions) that would influence the development of Norwegian livestock farming, and public and private animal health and AMR governance capacity. The main threats were identified as erosion of trust impacting a culture of organisational collaboration on animal health, loss of capacity and solidarity in the context of declining farmers and veterinarians, and the tensions this produces between winners and losers. This was the basis for identifying several actions including the development of strong local networks of farmers, integrating veterinary and farm advisory services, utilising Ai and data technology to improve national animal health monitoring, and the need for sustaining the institutional and economic structures that are pre-conditions for work on AMR and animal health. These results highlight the importance of attending to these broader structural and institutional conditions that facilitate or hinder the adoption of biosecurity, antibiotic stewardship and preventive veterinary health measures as industry stakeholders and public authorities in Europe continue to grapple with AMR and antibiotic use in livestock farming.

## 1 Introduction

Antimicrobial resistance has been a high priority issue in Norway, the EU and globally, for over a decade. There has been a significant renewal of international actions on AMR, especially since 2015 [see European Commission (1); Norwegian Ministries (2); World Health Organization (3)] to both reduce antibiotic use and minimize the selection and transmission of AMR bacteria. Adopting a One Health perspective, AMR governance has developed so as to take seriously the interconnections between animals, humans and environments (1, 4). Consequently, a core element of these efforts has been to directly address AMR risks that might arise through antibiotic use in livestock farming. This has been acheived through initiatives promoting responsible antibiotic stewardship and AMR surveillance in agricultural animals to reduce antibiotic use, and minimise the risk of AMR developing and spreading (2, 5, 6).

Governing AMR in livestock farming in Europe has principally focused on achieving antibiotic use reductions through ‘rational antibiotic use’ and surveillance of AMR prevalence through both targeted monitoring of key AMR bacteria of concern and sentinel species. However, AMR governance is an open-ended, multi-level process. That is to say that AMR is an evolutionary phenomenon that cannot be ‘fixed’ but is the constant development of microbes that requires ongoing governance action (7) to manage newly emergent challenges. Similarly, the capacities of governance are (re-)shaped by a diverse range of drivers within agriculture, veterinary sciences and society that include technological, environmental, political, social, and economic developments. Addressing AMR effectively and sustainably therefore requires planning with the future in mind (8, 9). This is particularly important in the context of Norway where the government has committed to a 10-year strategic planning cycle on AMR (10), in contrast to the previous 3–5 year policy initiatives (2, 11).

Scenario planning in relation to AMR challenges, including human and animal health, has been one approach that has aimed to develop strategies largely in the context of improving AMR governance in the context of diverse future conditions in different national contexts such as Sweden, Scotland, India and South Africa (8, 9, 12–14). Scenario planning is often used when numerous uncertain and unpredictable factors influence an outcome, as is the case with AMR (8). This study was based in Norway, a stable, high-income country which has achieved very low antibiotic use and low AMR prevalence in livestock farming. The challenge for Norwegian stakeholders, is that the current governance of AMR is considered effective and therefore desirable to maintain. Thus, our study explored possible future changes and challenges, as well as the possible strategic actions that would help maintain existing AMR governance capabilities and outcomes.

Norwegian authorities and notably, the agricultural industry, have taken proactive action to govern antimicrobial resistance risks since the mid-1990s. This has involved strict antibiotic regulation, animal import controls, antibiotic reduction targets, targeted initiatives to address specific areas of high antibiotic use, AMR surveillance, knowledge service coordination to support disease prevention and biosecurity measures, and MRSA and ESBL control programs (15, 16). This regime has been negotiated and coordinated through a collaboration between industry actors and public authorities. The result has been the achievement of the lowest sales of veterinary antimicrobial products for food producing animals adjusted for animal population (mg/PCU^1^) amongst the 31 EU and EEA nations in 2021 (17), and a low prevalence of AMR bacteria (16).

However, AMR is an open-ended challenge. Norwegian actors must therefore work to sustain existing capacities and systems of AMR governance, whilst remaining open to developing new strategies in response to both emerging AMR challenges and societal changes, including shocks, that might destabilize the current governance system and its foundations.

The aim of this study was to engage with a diverse group of Norwegian stakeholders and experts, through a scenario planning methodology to explore alternative futures and the actions needed to successfully maintain existing governance capabilities and outcomes in Norwegian livestock farming, under changing national and global conditions to 2050. The overarching goal was to inform the future long-term strategy for AMR surveillance, animal health and antibiotic use governance in Norway.

## 2 Materials and methods

### 2.1 Scenario planning

Scenario planning is a participatory methodology that has been widely utilised in a range of contexts and topics including business planning (18), science and technology governance (19), environmental management (20), animal disease surveillance (21) and AMR (8, 9, 12). At its core, scenario planning approaches involve exploring the future of society and its institutions so as to formulate strategies for action in response to different imagined outcomes (22). Increasingly they are used to bring together stakeholders and multi-disciplinary experts to enable integrated analysis and create meaningful results from scenarios. Scenarios in the context of this study are understood as constructed future narratives that reflect possible, but not necessary probably future states. They are not rooted in predictability, but rather an assessment of both the drivers of change and possible plausible alternative states that could result from them, within the context of interest (23).

Scenarios are therefore a narrative tool to enable participants to anticipate diverse future outcomes, challenge their preconceived assumptions and expectations about the system at risk, the challenges to it and possibilities for action (21). Scenario planning is particularly relevant in the context of wicked problems such as AMR, where probabilistic risk assessments and quantitative modelling have difficulty accounting for the complexity of influential interactions, high levels of uncertainty and subjective judgements that are important to incorporate in foresight processes. Such factors might include dramatic shock events such as COVID-19, climate change, changing public attitudes to AMR risks and/or advances in diagnostic technologies. The approach followed in this study combines elements from accepted scenario planning methodologies and applies them to the case of AMR in the Norwegian agricultural context.

Phase 1 involved preliminary data collection and analysis to: identify historical trends, policies and drivers to establish the baseline context; identification of relevant human health, animal health, agricultural, and environmental stakeholders; establishing the scope and future timeframe; identification of key change drivers, risks and events (social, economic, political, environmental, agricultural and biological); identification of key uncertainties. Key drivers were utilised to produce change axis around which alternative scenarios were sketched. Phase 2 involves synthesising and prioritising this data and scenario sketches to construct initial scenario narratives and themes, establishing plausibility and internal consistency of scenarios, and finally using them as decision support tools to develop strategies.

### 2.2 Data collection

The scenario planning methodology used in this study involved a three-step process.

Phase one involved interviews with 21 stakeholder representatives from the agriculture industry, public authorities, public health and veterinary science organisations. Potential participants for interviews and the workshops were identified through existing networks and knowledge of actors and individuals relevant to the aims of the study, followed by snowballing from there.

These interviews covered four topic areas: (1) history of AMR and its governance in Norway, (2) perceived important aspects and risks shaping the prevalence and experiences of AMR, (3) key drivers and governance measures and their justification, (4) future trajectories of change both with regards to drivers of risk and processes of governance. The aim of these interviews was both to develop an initial list of key drivers to be considered by the participants in the scenario planning workshop, and to provide important context and information for detailing the scenarios. Interview participants were invited to participate in the workshops.

Phase two involved two, one-day workshops that sought to develop and consolidate the future scenarios. The first scenario development workshop was held in June 2022 in Trondheim, the second ‘sense-checking’ workshop was held in November 2022 in Oslo. Participants were offered compensation for their time. 11 participants (five women, six men) representing veterinary, industry, retail and research organisations were assisted by five facilitators in the scenario planning activities. The focal question of the study was agreed as follows: What will the Norwegian livestock industry look like in 2050, and how can we maintain today’s system of management over AMR, antibiotic use and animal disease in the face of future challenges, opportunities, and changing conditions? AMR governance was considered in relation to three main areas: surveillance, animal disease prevention and biosecurity, and regulatory and policy framework.

In the first one-day workshop participants worked in two independent groups through a set of exercises that resulted in the creation of four scenarios describing the situation in 2050 for Norwegian livestock farming and the AMR governance system. The first activity was a priming exercise to develop a historical timeline outlining key developments in agriculture, animal health and AMR governance. The goal was also to allow participants to consider the dynamic nature of past changes and their drivers. The second activity was the scenario development exercise. Participants were presented with 51 physical cards, each with a different change driver and short description providing elaboration. The cards were developed based on interview data and covered change drivers linked to society and politics, technology and knowledge, economy, environment, demographics. The cards were reviewed, added to by participants, sorted, and then prioritized by each group, first on significance of impact (e.g., whether a driver was considered to have a high or low impact on future change) and then secondly on uncertainty (e.g., the degree to which a driver was considered to have multiple possible and plausible outcomes or a single plausible outcome). The high impact, high uncertainty drivers were then grouped into themes, with the group considering the cards and their previous discussion pertaining to each card. The resultant themes became the critical drivers around which scenarios were developed.

However, uncertainty does not directly translate into Norwegian without challenge. Discussion prior to the workshop and on the day was focused on communicating the intended meaning of uncertainty in this context. Two alternatives were discussed, (*u*)*sikkerhet* which also means (in)security, and (*u*)*sannsynlighet* which means (im)probability. In the end all three were used to ensure clarity of meaning, but as we discuss later, a probabilistic understanding of the uncertainty of the future dominated.

Three critical drivers were the same for both groups: (1) government prioritisation of agriculture and AMR (high versus low), (2) Norway’s response to global events (open versus protectionist) and (3) Trust in government (high versus low). Alignment between EU and Norway was also considered, however this was with regards to policy alignment not membership. These were combined with three high impact, low uncertainty drivers (farm and veterinarian demographics, structural change, and technological change) to produce four total scenarios (two per group). A preliminary scenario narrative was characterised by participants, guided by a set of key questions that prompted the groups to discuss and agree the scenario endpoint conditions, and the key events that would be required to produce the scenario endpoint. Scenario development was guided by plausibility, internal consistency and logic and an attempt to produce diverse futures that would stimulate discussion.

The axis led to the choice of four scenario spaces explored by participants:

- *Scenario 1*: Moderate prioritisation of agriculture, Moderately protectionist, High trust
- *Scenario 2*: Low prioritisation, Moderately open, Low trust
- *Scenario 3*: High prioritisation, Moderately protectionist, High trust
- *Scenario 4*: High prioritisation, Highly protectionist, High trust

The axis, in theory, provided space to develop unique scenario spaces. However, in practice the group discussions were heavily influenced by an underlying commitment from participants to the Norwegian model of agriculture. This involves import restrictions, market regulation of certain agricultural commodities, extensive industry-state collaboration, and production and non-production subsidies. Similarly, major global disruption was interpreted via the lens of the ongoing experience of COVID, its disruptions and restrictions. Norway, and the agri-food system, had weathered this relatively well in comparison to neighbours and other European countries, due to the (re-)emphasis on local and nationally produced food. Consequently, the axis extremes were interpreted within the context of this system and recent experience, which reduced the scope and scale of anticipated changes in the agri-food system and for AMR governance arrangements. Scenarios thus reflected a “more/less or better/worse rather than either/or logic” due to a lack of clear cut distinctions between one future state and another (24, p. 547).

The four preliminary future narratives were initially developed further by the researchers following the end of the first workshop to add detail and depth to produce extended scenario narratives (see Supplementary materials). The extended narratives were developed based on the notes taken on the day by facilitators and through incorporating insights from the preliminary interview data. This helped to deepen the description, further clarify the underlying assumptions, and detail the endpoint and key events description to produce a coherent narrative for each scenario. The extended scenario narratives were re-circulated to the participants for individual input and comment. However, the four scenarios had a relatively low scope for change, reflecting what participants considered to be plausible in the Norwegian context. There was significant consistency and overlap between the scenarios produced by each group, despite both groups working independently.

The second one-day workshop involved presenting the extended scenario narratives to the same stakeholders once more to ‘test’ the plausibility of the scenarios. Although the four scenarios were initially considered separately, the stakeholders’ views of the future were highly convergent. Scenarios 1 was considered the status quo scenario, 3 and 4, explored ‘optimistic’ futures where trust in government is high, agriculture is prioritised, and Norway is protectionist in its approach to global shocks and developments. Whilst scenario 2 was considered by participants to be pessimistic. Furthermore, the scenarios were considered to raise a similar range of strategic questions for AMR governance. Stakeholders were encouraged to consider more radical alternatives, but these were not considered credible. Consequently, and in response to feedback from participants and facilitators during the workshop, we combined the scenarios into a single ‘core’ future vision to 2050, whilst retaining some openness for different possibilities in respect to key change variables. When developing the core vision from the four scenarios, we prioritised retaining the key dynamics that contribute to different challenges and opportunities highlighted from across the scenarios, and to reflect a broad spectrum of potentially relevant issues identified from the first workshop. We will reflect on the consequences of these decisions later in the discussion/results. The workshop therefore returned to the six key change drivers and developed them into short vignettes that described how they would develop over time towards the 2050 vision.

Phase three involved a one-day strategy development workshop held in Trondheim, June 2024. Prior to the workshop, several smaller digital meetings were held with the same stakeholders to discuss key topics raised by the two earlier workshops. The participants worked with the vignettes and the 2050 vision to stimulate discussion about the strategic challenges that the anticipated changes posed to the continued effective functioning of AMR governance in Norwegian livestock farming. The aim was to prompt a set of strategic questions regarding the future organization, functioning and resourcing of AMR governance in Norwegian livestock farming in 2050. Working independently in two groups, participants were first asked to reflect on critical factors supporting the current system, and its anticipated resilience in relation to the imagined impacts of the six change drivers. During the lunch break the researchers worked to translate the outcome of this discussion into a set of concrete strategic questions, each of which linked to a key challenge identified through the group deliberations. For example, how can actors bridge the loss of veterinary capacity, especially in more isolated areas? How to maintain collaboration between industry actors in the context of growing market competition between them? In the final activity, we used the questions as a means of developing a set of strategies that were considered to have broad utility and robustness in the face of future uncertainty and anticipated challenges.

### 2.3 Study limitations and reflections on research design

The type and plausibility of the future narratives and subsequent strategies developed within this participatory process were influenced by several factors: diversity of participants, power dynamics within participant groups and between participants and facilitators and resource constraints, including time and participant availability. Notably, workshop participants who agreed to participate in the study were primarily from Norwegian veterinary and agricultural sectors. We did not explore deeply the reasons for agreeing to participate, but on reflection, this is a small and well-connected sector, and the use of convenience sampling to recruit participants through researcher networks, the potential emphasis on institutional actors and the recognition of the lead organisation and investigators may have influenced participant diversity and discussion outcomes (25). Other participants from civil society, public health and environmental health sectors were invited but could not attend for various reasons. The latter perspectives were important and attempts to strengthen and diversify the scenario development process were made by including information obtained through interview data. The final future vision and resultant strategies therefore, primarily reflected the priorities and preferences of existing agricultural organisations, and may not necessarily reflect those of a broader spectrum of interested stakeholders who might also have a say in shaping AMR governance debates and policy in Norway.

As scenario narratives emerged, it was clear that three out of the four initial scenarios were converging towards a single vision of the future characterised by prioritisation of agriculture and a protectionist approach to governance. The nuanced aspects of building and sustaining trust were considered separately in this future (drawing on elements of both low and high trust futures). Alternative futures, which considered possible events, such as Norway’s ascension to the European Union as a full member state, could not be developed into cogent scenarios because they were not considered plausible within the timeframe under consideration by the assembled participants. Although EU membership could have been a ‘false distinction’ [see Roth et al. (24)] given the degree of existing alignment between Norwegian and EU policy, including in areas of AMR and animal health. Nevertheless, the participatory process did create space to challenge and examine diverse participant’s perspectives and assessments on the future vision, and its implications for agriculture and AMR governance.

The emergence of a single vision from a foresight exercise is not in itself inherently problematic, as long as the process is transparent, relevant to and led by participants, and has a consistent internal logic (8, 26). However, Duckett et al. (25) argue that when “participatory stakeholder engagement is brought into the policy development arena it demands the highest level of accountability, absent in parallel exercises conducted in the corporate world… it is therefore under a special obligation to be self-critical.”

Facilitation plays an important role in unearthing hidden assumptions and enabling participants to explore potentially uncomfortable futures which may be plausible but not necessarily likely nor desirable. However, it takes place within a pragmatic context in which the need to ensure effective engagement, progress the scenario process, differences in cultural and professional positionality, and limited available time, all shape the outcomes. Consequently, facilitators sacrificed some directionality in practice, which can inadvertently reinforce and perpetuate existing biases and stereotypes of the future resulting from power imbalances which are difficult to overcome (25).

It would be beneficial to build on this work in future, by engaging consumers, farmers and veterinarians directly. Representatives from the private sector in agriculture and retail did participate, but perhaps this introduced an institutionalised perspective and obscured or mitigated opportunities to draw out personal viewpoints. An additional option for breaking participants out of established ways of thought could include working with scenarios generated by other groups or individuals, including from previous research, and separate from the stakeholders directly relevant to AMR governance. Such an approach would have required participants to respond to said scenarios and changes that are in effect outside of their control to determine.

Pragmatically, adapting towards a single vision approach was also a means of navigating the high degree of consistency between the scenarios, whilst trying to develop a productive narrative tool with which stakeholders could think about the existing resilience and future challenges for AMR governance. The decision not to press for the introduction of new future considerations, especially those that broke with the drivers and assumptions established earlier, was rooted in a desire to retain a narrative that was considered credible and valid by the participants and had emerged from their own deliberations. Especially important given the overarching aim was to use scenario planning as a mechanism to contribute to long-term strategic planning in this governance domain by working with stakeholders, and not simply to produce scenarios as an outcome in and of themselves (27).

Furthermore, this outcome reflects that there is a long history of close collaboration between state and industry actors in this area. AMR governance is part of a broader system that has high organisational capacity to deliver interventions in practice, and a collaborative culture involving national membership organisations, such as cooperatives and farmers unions, that has proven highly effective at disciplining different actors towards enacting dominant discourses (28). Arguably, these pre-existing networks were collectively shaping future imaginaries prior to the scenario exercise as well as what was considered plausible. Ultimately, the four original scenarios were constrained by the worldviews of the participants and the research process that attempted to reduce the number of considerations to a small number of high impact, high uncertainty drivers. This likely contributed to creating the conditions in which the scenarios trended towards a consensus position. This also reflects broader power dynamics within the sector that although external to the scenario planning process were articulated within it, and which exist irrespective of the number of scenarios being produced. These are uncontrollable preconditions that can only be acknowledged (25), so that strategic guidance does not originate from a black box (26).

## 3 Results

The results reported in this article draw from across the three workshops, with an emphasis on the development of the core vision (outcome of workshop two) and its use for the development of strategies (outcome of workshop three). The section is organized as follows. Section 3.1 presents a short summary of the historical context outlining key past structural, political, economic and social dynamics that shaped the Norwegian livestock sectors and AMR governance. Section 3.2 details the six critical change drivers identified in the first workshop and used to form the outlines of the initial scenarios by the participants. Section 3.3 provides a description of the future vision of Norway in 2050, which resulted from the second workshop. This vision provided the basis for identifying the key challenges, opportunities and strategies which are outlined in detail in section 3.4.

### 3.1 Historical context

The development of the historical timeline identified important past events and influences regarding Norway’s livestock sectors and AMR governance. The timeline was broken by participants into four broad periods, each considered to have redefined core conditions.

#### 3.1.1 1890–1940: the formative years of the Norwegian state

Establishment of key institutions included the Norwegian Veterinary Institute, the Norwegian School of Veterinary Science, and the development of the Norwegian agricultural cooperatives. In 1928, Norway established one of the earliest pharmaceutical regulatory systems (29). The 1938 Pharmaceutical Import Act (Lov om innførsel av apotekvarer og gifter) introduced the ‘need clause’ which required that a drug must be medically needed or significantly improve on an existing drug. It restricted the number of drugs available on the market in Norway in comparison to other European and Nordic nations (30). The system was cited as extending the regulatory space of the state over antibiotics, comparative with other countries (29).

#### 3.1.2 1941–1980: the development of the post-war social democratic order

Agriculture received considerable support and protection from the state and underwent a significant technological and structural transition similar to other parts of Europe. Livestock numbers declined, notably, the number of dairy cows fell from 807,000 in 1950 to 391,000 in 1980, and production volumes increased, for example the annual pork production increased from 48 thousand kg in 1959 to 78 million kg in 1979 (31, 32). Simultaneously, the number of farm holdings declined by around 50% from 200,000 in 1959 and to just over 100,000 in 1979. Antibiotics entered agriculture in the early 1950s and in addition to disease treatment international pharmaceutical companies and feed producers began marketing antibiotic amended feeds to produce a growth promotor effect. In 1975, a farmer’s protest, the Hitra Action, called for improved prices for meat and milk and social conditions for agriculture (31). It received significant national attention and resulted in the government agreeing to improve farmers’ incomes to become comparable with industrial workers before 1982.

#### 3.1.3 1981–2000: EEA ascension and an industry led antibiotic reduction programme

An aquaculture disease crisis saw antibiotic drug use in the industry rapidly increase (33, 34), peaking in 1987 at 48,000 kg (35). Although resolved through the development of salmon vaccines, (35), public controversy about the risk of AMR from high antibiotic use continued. This pressure intensified when in 1993 an association was observed between the use of the antibiotic avoparcin in poultry and vancomycin resistance (36–38).

In 1994–95 the industry initiated the “Friskere dyr og mindre bruk av antibiotika” (Healthier animals and less use of antibiotics) project that aimed to improve animal health and lower antibiotic use by 25% by 2000 (baseline 1995) (15). It included a voluntary ban on the use of antibiotic growth promoters, disease prevention measures to reduce therapeutic antibiotic use in livestock and restrictions on group prophylactic treatment. Veterinarians were given a central role working with farmers to achieve improvements. By 1999 antibiotic use had fallen 40% against the 1995 levels (39). Disease resistance was given greater priority in the targeted breeding values adopted by the breeding cooperatives Geno (Norwegian Red) and Topigs-Norsvin (TN70 pig).

In 1992, the EEA Agreement was signed, entering into force in 1994. Norway retained control of agriculture, customs and trade policy, but aligned with the EU. The previous political arrangements supporting agriculture, were in flux. The result has been a mix of liberalised internal market conditions, strong trade protection, farmers receiving high financial support and cooperatives remaining important actors.

#### 3.1.4 2000–2022: a shift towards state-led AMR governance

In 1999 the Norwegian government published its cross-sectorial Action plan to combat antibiotic resistance (2000–2004). The action plan established the NORM-VET surveillance programme, coordinated by the Norwegian Veterinary Institute, with the aim of monitoring antimicrobial resistance in bacteria from feed, food, and animals. This is reported in the annual NORM/NORM-VET reports, together with the surveillance of antibiotic sales in food producing animals, and data regarding the AMR situation in the human medicine. This was followed by a 2008 National strategy for prevention of infections in the health service and antibiotic resistance (2008–2012) that sought to maintain the favourable situation in Norway, whilst signalling a greater emphasis on MRSA (and ESBLs as an emerging concern) nationally (40).

Norway’s 2015–2020 AMR strategy followed growing momentum at UN and EU-levels for coordinated action on AMR. The strategy set a 10% antibiotic use reduction target for agriculture (baseline 2013) and committed to two already initiated programmes, (1) preventing MRSA from being established in swine, and (2) reducing the prevalence of ESBL *E. coli* in poultry to a minimum (2). The latter was led by the Norwegian Food Safety Authority, the former by the poultry industry. Animalia coordinated an industry action plan to support realising these objectives (15).

Structural changes continued. Farm numbers fell from 99,400 in 1989 to 37,600 in 2022 (41). The average farm size increased from 14.7 ha in 1999 to 26.1 ha in 2022. This was accompanied in a large increase in the amounts of land being rented. Animal numbers remained largely stable for sheep, pigs and cattle overall, although milk cows continued to decline, offset by an increase in beef cattle (41). Poultry production saw the most significant growth with annual broiler chicken production increasing from 3,2 million birds in 1989 to around 16 million in 2022 (42).

### 3.2 Future change drivers

Six change driver vignettes were developed to describe the developments between 2025 and 2050 that would lead to the 2050 vision. They aimed to capture key changes identified through the scenario process that had strategic consequences for considering the future resilience of the Norwegian livestock AMR governance system.

#### 3.2.1 Farm centralisation and up-scaling

Despite political contestation of the level of state support to agriculture, the core policy framework which favours modernisation, consolidation and centralisation, has not been destabilised. Regulations requiring dairy farmers to rear animals in loose barns by 2034 were extended to give farmers more time, but still resulted in a large exodus of dairy farmers who chose not to invest. Fewer, larger farms that are more geographically centralised has been supported by gradual reforms to the subsidy and quota regime to support and incentivise larger herds. These developments have seen the demographics of farmers move slightly older but with the loss of many part-time farmers. High financial demands for taking over a conventional commercial farm have seen new entrants primarily move towards smallholding and engaging in direct local markets outside of the mainstream veterinary-slaughter-processing-retail system.

#### 3.2.2 Declining veterinary numbers and coverage

Access to veterinary services, already a problem in 2020s continued to increase, especially in more remote areas. Efforts in the late 2020s and early 2030s to improve retention by offering better economic support for vets was largely unsuccessful as it could not address key quality of life issues linked to the lonely and challenging nature of the work. Vet numbers has therefore mirrored broader structural change. Coverage remains good in central livestock areas where fewer, larger units mean a better economy and quality of life for vets due to less distance between large farms that have multiple assignments. Routine and emergency access in peripheral and remote areas has however become a major problem. In the public sector, the Norwegian Food Safety Authorities is increasingly reliant on digital solutions and data analysis to identify threats because in person visits are too resource intensive. Although data collected from large commercial farms is of high quality, many smaller farms fall out of formal reporting systems. Gaps have therefore emerged in veterinary and data coverage.

#### 3.2.3 Cooperatives and supermarkets new balance of power

Falling farm numbers resulted in declining membership for the cooperatives Nortura (meat and eggs) and Tine (milk) dominated by professional commercial farmers committed to modernisation and up-scaling. Simultaneously, supermarkets have continued to aggressively push their discounted own product labels at the expense of cooperative product lines. Cooperatives have therefore found themselves caught between intense market competition and a policy role to deliver target farm gate-prices. Nortura responded by splitting its activities creating a slaughter and wholesale segment, and product production and marketing segment. The latter of which collapsed in the late 2030s. Tine and Nortura sought to secure their position through developing supply partnerships with supermarkets. However, supermarkets are now able to significantly steer the priorities and practices of other agri-food sector actors through these arrangements.

#### 3.2.4 Climate change, disruption and emerging and re-emerging diseases

Climate change impacts, especially an increase in extreme weather events continue to increase in intensity. Damage and disruption to agricultural crops and grazing land, farm and non-farm transport and communication infrastructure has become more severe. More broadly, changing climate has increased risk from emerging and re-emerging disease and resulted in periodic outbreaks. Once eradicated production diseases have re-occurred in sheep, cattle and poultry, contributing to a gradual increase in antibiotic use. Prioritisation by industry and public authorities to re-establish disease free status and recover from extreme weather events has strained resources and capacity. The toll of recovering from extreme weather events and disease control measures has contributed to farmers leaving and the reduction of active farms.

#### 3.2.5 Resources, infrastructure and expertise: multiple policy priorities

Ongoing global volatility has had mixed implications for Norwegian society and agriculture. Continued oil production, a pivot towards renewable energy, mining and fish exports mean Norway continues to have a relatively strong economic and institutional position. Food security has remained a priority resulting in the maintenance of relatively generous support to farmers despite other priorities. However, public authorities and industry organisations operate within a resource constrained space with multiple competing priorities. Digitalisation and big data analysis has been adopted as a solution for monitoring animal health situation at the farm and sector level (e.g., analysis of national animal health service data). The availability of resources for AMR surveillance, including infrastructure and expertise, is tight and in constant competition with competing needs, especially emerging diseases and animal welfare. This is compounded by low antibiotic use and AMR prevalence making it easier to prioritise other seemingly more urgent issues.

#### 3.2.6 Trust in institutions

Conventional commercial farmers are well-integrated and supported by both public authorities, national cooperatives and private services. Their trust in key institutions remains relatively high and they are well engaged with formal organisations and political negotiations. This contrasts with smaller farmers and those operating in more isolated areas that have faced increasingly poor access to markets, services and veterinary advice. A feeling of abandonment has produced distrust in state authorities and industry organisations. These farmers are organised locally and nationally outside of established farmer cooperative networks, often through social media, and are hard to reach and engage with as a result.

### 3.3 Vision: Norway in 2050

#### 3.3.1 Norway in 2050

In 2050, Norway remains part of the European Economic Area and retains control over agricultural and trade policy. Maintaining domestic food production remains a high political priority. Farming has been affected by challenging market conditions and variable state fiscal support. Farm numbers have fallen 50%, and the sector has structurally polarised between larger, family-run commercial farms concentrated in the most viable regions and dispersed small-scale part-time and hobby farmers. Larger farms are often reliant on employed workers with increasing numbers coming from outside of Europe. National livestock numbers declined to 2040 before stabilising. A veterinary recruitment and retention crisis has been managed through a shift to digital farm visits and mobile veterinary practices. But the loss of local networks has impacted state capacity as resource constraints saw the Norwegian Food Safety Authority increasingly rely on private vets to assist in handling disease outbreaks. Supermarkets have become the key actors in the Norwegian food system at the expense of the national farmer cooperatives, Nortura (meat and egg) and Tine (milk). Supermarkets have used their position to determine priorities and requirements through partnerships with other actors. An increase in trade, travel and work migration with non-EU countries, and climate change have all contributed to an increase in the likelihood of spread of endemic and exotic animal diseases, such as African Swine Fever, Bluetongue virus, bovine ringworm, bovine viral diarrhoea and sheep scab. Larger farms have generally good biosecurity controls, disease surveillance and management capacity. Good access to capital has allowed investment in technology, sensors and software for disease management. There is more limited capacity on small-scale and lower income farms. Industry operated health services coordinate advice to farmers on disease prevention and collect data on antibiotic use and disease incidences. The quality of data is high and there is high certainty about the prevalence of disease amongst different sectors. But some farmers feel abandoned by state authorities and alienated from industry organisations, leading to a breakdown in trust that has impacted organised disease control and monitoring programmes. Low trust has also contributed to gaps in data coverage due to low submission rates to veterinary laboratories and reporting databases. There remains pressure for the agricultural sector to maintain low antimicrobial usage, and AMR prevalence is monitored nationally. Farmers and veterinarians remain committed to low antibiotic use. However, maintaining the resource intensive MRSA surveillance programme in pigs has limited the resources and capacity to expand surveillance to emerging organisms of concern.

### 3.4 Challenges, opportunities and strategies

The current system of animal health and AMR governance in Norway is characterized by a culture of state-industry collaboration and cost-sharing. The Norwegian government is responsible for resource prioritization and negotiating framework conditions with the farmers unions. The Ministry of Agriculture and Food is responsible for policy, whereas regulation and supervision of animal health, welfare, food safety and imports are the responsibility of the Norwegian Food Safety Authority. These are supported by a state-financed research infrastructure that includes the Norwegian Veterinary Institute.

AMR and animal health policy making and implementation involves close collaboration with a range of industry stakeholders. These are all national membership organisations covering the whole of Norway. Principal stakeholders are the four farmer cooperatives [*Tine* (dairy), *Nortura* (meat and poultry), *Geno* (dairy breeder) and *Norsvin* (pig breeder)], the two farmers unions (*Norges Bondelag* and *Norsk Bonde- og Småbrukerlag*), and four industry associations [*KLF* (meat and poultry industry), *Norsk Sau-og Geit* (sheep and goat producers), *TYR* (beef producers), *Norsk Fjørfelag* (poultry)]. Producer cooperatives such as Nortura and Tine are key governance actors responsible for managing target prices and production quotas on behalf of the state. Whereas the farmers unions are involved in annual negotiations with the state to set the overall subsidy framework, its priorities and levels of economic support, and therefore broadly speaking, farmer incomes.

Nortura and KLF own Animalia which is a knowledge and competence organization supporting meat and egg farmers and operating the industry-led health services. It is a key node organization. The three dominant grocery chains (Reitan, NorgesGruppen, Coop Norge), by market share, are not currently involved in directing animal health and antibiotic use priorities to the degree seen in other European countries. Instead, they have supported state-led, industry wide quality assurance system (Kvalitetssystem i landbruket). One exception is Reitan, which has taken over the poultry meat company Norsk Kylling and developed a new production and animal welfare marketing approach around the slower growing Hubbard breed in alliance with the animal welfare organization Norwegian Animal Protection Alliance (Dyrevenalliansen).

The strategies discussed are therefore anticipated as being implemented within the context of this broader governance framework with distributed responsibility and close collaboration between state and industry.

The vision and the key trends were used to identify risks that would lead to less effective AMR and animal health management and surveillance, and opportunities to improve delivery. The resultant strategies where clustered into the following topic areas:

1. Building solidarity – strategies to build and maintain trust and collaborative working arrangements between actors,
2. Building competence for the future – strategies to support the development of farmer and veterinary competences for AMR and animal disease prevention, monitoring and control,
3. Restoring the foundations – strategies to ensure the core economic framework for vets and farmers is improved to limit capacity loss.

#### 3.4.1 Building solidarity—strategies to build and maintain trust and collaborative working arrangements between actors

A future in which farmers are polarised between centralised, large-scale commercial farmers and dispersed small-scale part-time and hobby farmers was situated as threatening the collaborative model that has been successful in Norway. Large-scale conventional farmers were expected to remain strongly integrated with formal structures, and have good interaction with local veterinarians, industry initiatives and cooperatives. Small farmers were positioned as increasingly rejecting and falling outside of these networks, in favour of developing their own social media mediated advisory and support networks. This was compounded by the loss of veterinarians, expected as occurring to a greater degree in more peripheral and isolated areas, eroding further the links these farmers might have to support and reporting systems. Furthermore, if small-farmers ‘drop-out’ of the system, it risks producing a highly incomplete picture of animal health, medicine use and AMR, whilst eroding capacity to enact new education and control initiatives. This could create greater opportunities for the circulation of AMR bacteria of concern or emerging animal diseases ‘under the radar’. In sum, these developments risked fragmenting the system, threating political solidarity and practical cooperation on AMR and animal health prevention and control.

*Strategy*: developing competence in social media communication to reach isolated farming groups

- Norway has a long history of successful communication and working across the industry-policy-science nexus and this was expected to continue. However, the link between this nexus and farmers was at risk due to the changing composition of farm structure and farmer practices.
- Current strategies are based on well-established channels of communication through cooperative and union networks, assume a particular audience of conventional farmers, and do not have particularly sophisticated engagement with social media networks.
- Social media has become an alternative forum through which information and networking are taking place in Norwegian agriculture.
- Animalia is best placed to develop new communication channels and competence to reach different audiences. Given resource constraints this would involve developing connections with existing social media influencers to promote the circulation of knowledge and information to promote responsible practices. A secondary objective is to maintain solidarity between farmers that have and have not experienced a disease threat, but where there is a need for collective action.

*Strategy*: local action network building to secure solidarity and a basis for collective action

- Multi-level cooperation and networks were critical to past success on animal health and AMR, but were imperiled by both the changing structural conditions, loss of veterinarians and farmers, and growing distance between large and small producers.
- The need to develop strong local networks was not just linked to disease prevention and work on AMR but also to reduce the risk of social isolation. Local networks would work to build solidarity and cooperation with regards to specific local challenges, especially where voluntary actions are required, such as local limits on animal trade, vaccination, and the adoption of new hygiene measures.
- These networks would build upon existing arrangements and state-industry collaboration. Farmers unions in cooperation with Animalia are key actors who have the capacity to mobilise local action networks through existing structures. Depending on whether the problem is the statutory responsibility of the Norwegian Food Safety Authority or voluntary action will shape the lead actors in specifying the scope, resources and types of measure.

#### 3.4.2 Building future competence—strategies to support the development of farmer and veterinary competences for AMR and animal disease prevention, monitoring and control

Although there was considerable optimism about the opportunities posed by new technologies, such as animal side diagnostics and sensors, and new data analysis and management opportunities through big data and artificial intelligence, there is no coordinated policy to support farmers in effectively utilising them to maximise their potential. Training and education for technology use is handled by technology companies, often multi-national companies that provide generic advice not tailored to Norwegian regulations, and who have no contact with other advisory authorities. Simultaneously, a loss of farmers and veterinarians was considered to present a twofold challenge. First, the loss of accumulated intergenerational knowledge about animal disease, medicine use and AMR. Second, the reduction in size of farmer and veterinary networks, results in the loss of formal and informal consultation with groups, leading to a loss of capacity and collective knowledge. This is in a context where there are increased knowledge demands of modern farming, particularly as welfare, sustainability and management requirements grow, but overall, a declining number of advisors (not just veterinarians). Although there are less farmers that need to be served by advisory services, the geographically distributed nature of farming increases the proportion of herds that are likely to be ‘hard-to-reach’ and therefore miss out on support, training and information.

*Strategy*: creation of a consolidated advisory service to align advice with strategic priorities

- Advisory support is fragmented between private veterinarians, cooperatives’ advisory services, the Norwegian Agricultural Advisory Service, and a growing array of private technology providers. A consolidated advisory service was positioned as one mechanism for bridging both a need for diverse types of advice and creating opportunities for integrated advisory provision.
- This is especially important given that a growing number of health management technologies are being developed, but equipment suppliers are responsible for training, much of which is not tailored to Norwegian regulatory or production conditions.
- More integrated advice on issues related to animal disease prevention and control, medicine use and AMR that is tailored to new technology developments and other sorts of business management and sustainability advice.
- Organisationally, this could either involve an integration of existing animal health advisors from cooperatives into the Norwegian Agricultural Extension Service, which is composed of 10 regional extension units, or the establishment of an entirely new organisation.

*Strategy*: investment in new digital advisory solutions for cost effective monitoring

- Monitoring correct antibiotic use, monitoring of resistance, and monitoring animal health are all important for maintaining existing capacities. This is made more challenging as declining veterinary numbers reduce routine contact.
- Although centralisation and upscaling will make certain areas easier to monitor and contact, more isolated farms will potentially remain with poor veterinary support. This contributes to reduced opportunities for knowledge exchange and learning. However, Norway has relatively good internet services across most of the country, and a technologically competent society used to digital solutions.
- Such a model could make efficient use of limited veterinary, scientific and other advisory resources, whilst providing insight into what is happening on farms, individually and in an area.
- The use of digital solutions depends on the future organisation of veterinary sector. The Norwegian Food Safety Authority has already begun exploring the options for using digital inspections using farmer’s smartphone and tablet cameras. However, for private veterinarians it is dependent on farmers being willing to pay to access veterinarians and other advisors digitally rather than a physical visit. Digital solutions could be more viable in more isolated areas, or in the context of a consolidation of veterinarians into large regional units.

*Strategy*: investment in animal health education to sustain key competency amongst farmers and vets

- The loss of farmers and veterinarians risks eroding capacity and accumulated knowledge on animal disease handling and treatment. Furthermore, new entrants are not required to have an agricultural education, meaning that there are potential for gaps emerging in animal health education that previously were bridged through contact with local veterinarians. But in more peripheral and isolated areas, these contacts and networks are also being lost.
- A minimum competence and training requirement for animal rearing, animal health, correct medicine use, and antimicrobial resistance was suggested as a measure to ensure enhanced competence for all farmers. This could be in the form of a legal requirement for education or minimum competence, or a part of the KSL system and thus a requisite to not receive a deduction from the full price of sold animals.
- The additional educational requirement for veterinarians, delivered by the Norwegian University of Life Sciences for vets who have been trained in other countries, prior to being able to receive authorisation in Norway, was mentioned as a model.

#### 3.4.3 Restoring the foundations—strategies to ensure the core economic framework for vets and farmers is improved to limit capacity loss

Joining the European Union was not anticipated as plausible within the timeframe of analysis. Consequently, participants expected that Norway would remain committed to its model, which includes the four main high-level agricultural policy objectives (food security, maintaining agriculture across the country, increased value creation, and sustainable, low emissions agriculture), the legal and regulatory framework that seeks to limit market risks for farmers, and annual negotiations between farmers unions and the government over the level of fiscal support for agriculture. But current framework conditions were not averting the loss of farmers and veterinarians, whereas growing competition between cooperatives, private food processors and supermarkets were creating new instabilities and tensions. If this trajectory continued unaddressed it would erode both institutional and on-farm capacity, the social contract important for motivating action on antibiotic use and AMR, and the economic conditions that had enabled farmers to make investments in new technologies. A holistic approach to maintaining AMR and animal health governance therefore needed to engage with the foundations of Norwegian agriculture and ensure that framework conditions produced the political and operational conditions for effective collective action. Notably, maintaining capacity was being envisioned through the lens of maintaining the current networks of actors, their distributed responsibilities, and limiting capacity loss.

*Strategy*: investment in veterinary capacity

- Private veterinary coverage and industry veterinary capacity is necessary to maintain the whole system, not just on animal disease and AMR but also food safety and animal welfare. There needs to be greater effort to sustain this infrastructure.
- The state is the principal actor required to enact either of the two main options, (1) addition support for veterinarian incomes, subsidies for travel, grants and other incentives, or (2) re-organisation of the sector.
- Veterinarians could become full public employees of the county administration. A potentially attractive option in areas where the economic base is otherwise not sufficient to support a vet in private practice. This would give them additional work tasks but provide them with fixed employment and stable salary, which has been effective at retaining veterinarians in other parts of the animal health system.

*Strategy*: new animal health strategy

- There was a danger that the current good status of animal health and AMR in Norwegian farming becomes a cushion for de-prioritisation of animal health relative to other demands.
- The current Animal Health Strategy expires in 2025 and needs to be replaced with a much more active strategy that also re-establishes priorities for the next period and deals with emerging issues. A new strategy would be formally established by the Ministry of Agriculture and Food, through dialogue with industry stakeholders. Animalia would coordinate, with other industry stakeholders, the development of joint industry action plans to support implementation.
- Additional priorities would include: (1) animal movement, including the import of rare and exotic livestock animal breeds, and the movement of animals within Norway. Currently no good oversight of the sale and buying of animals, nor their movement, and it can contribute to the spread and re-establishment of production diseases that require antibiotic control. (2) Control and prevention of list-3 diseases needs to be given higher priority and active management. (3) Requirements for farmers to ensure good knowledge and competence in animal management and animal disease prevention and treatment.

*Strategy*: improve framework conditions for farmers

- Farmer economic conditions are an important precursor for other types of action, including investments in preventative animal health measures and influencing the likelihood that farmers will support new initiatives to achieve AMR goals that increase work burden. Similarly, compensation is part of the reciprocal nature of the system and underpins action on AMR and animal health. If it is eroded, then it presents real challenges to ongoing work.
- Framework conditions are the result of annual negotiations between the two farmers unions and the state, which sets the broader economic framework. It is important that the political will to sustain this is reinforced by farmers and industry actors.
- This requires effective political representation and negotiation by farmers unions and government willingness to both support farmers income. It also requires effective negotiation between farmers unions and both public authorities and private insurance providers to ensure adequate compensation when animals are culled to prevent and control disease.

## 4 Discussion and conclusion

The scenario planning workshop produced a plausible vision of the future for Norwegian AMR governance. This vision highlighted key drivers of change that would affect long-term capacity and the ability to sustain effective existing governance of AMR that had achieved both very low levels of antibiotic use and AMR prevalence amongst key bacteria of concern. Past success of AMR governance in Norway is the result of a high degree of organisational capacity to deliver antibiotic reduction, preventative disease and AMR eradication efforts in practice (28). This is rooted in a collaborative culture, a strong social contract, and high trust in institutions that have been highly effective pre-condition for implementation. The future vision was defined by uncertainties related to agricultural framework conditions, the balance of power between key food system actors, and tensions over resource prioritization. In short, the main threat is to this cultural and organisational system.

Although we began with the ambition of developing multiple different scenarios, the co-creation process quickly moved towards a single future vision due to a high degree of consensus across the stakeholders. This is arguably the result of close working and collaboration between state and industry actors in this area for nearly 30 years, meaning that pre-existing networks are collectively shaping future imaginaries prior to the scenario exercise, producing consensus within the scenario development processes. Consequently, two groups, working independently, developed very similar scenarios, based on the same set of drivers. Following review and credibility testing with the stakeholders we merged these into a single vision with a realm of plausible change, largely representing different degrees of change.

This provided a basis from which to develop strategies anticipated as mitigating risks and sustaining key capacity and enabling pre-conditions of good AMR governance. These strategies highlighted three core areas for consideration, (1) building solidarity to maintain trust and collaborative working, (2) building competence for the future in a context where sensors and animal data analysis become common mechanisms for disease management, and (3) restoring the foundations to ensure good economic capacity for investment and action, as well as the means for recovery.

These strategies were shaped by the problem definition that sought to support the existing Norwegian system and its collaborative arrangement that results in considerable resource and capacity sharing between the state and industry. An arrangement that has proven to be effective to date for achieving low antibiotic use, AMR prevalence and overall low levels of animal disease. It is perhaps unsurprising that this system retains support. But it does mean that strategies reinforced tendencies towards supporting established institutions, structural and power arrangements. Raising questions about what might be beyond the scenario, and what might this mean for long-term strategic planning.

The most notable absence from the scenario is with regards to Norway becoming a full EU member state. This was justified by participants on three grounds. Firstly, because plausibility was understood primarily through the lens of probabilistic considerations (see earlier discussion on translation). Thus, EU membership within the timeframe being considered was deemed highly unlikely. No major domestic political party is committed to full EU membership in the near term, and when polled it remains unpopular amongst the electorate (43). Secondly, because Norway is already aligned with the EU with regards to animal health and AMR policy (44), due to its membership of the EEA and coordination with the European Medicines Agency, it was not considered to significantly impact existing AMR initiatives and priorities. Joining the EEA in 1995 was considered a greater shock as it required major re-formulation of Norwegian legal and regulatory frameworks to ensure EU alignment across the whole economy. Thirdly, the main impact was considered in relation to agricultural structure and centralisation caused by changing regulation and subsidies, but this was positioned as an intensification of existing trends, e.g., greater degree of centralisation and up-scaling (45), which were already considered as part of the vision.

Furthermore, the strategies described here fall well within what might be considered the ‘mainstream’ imaginary of how to tackle animal health and AMR challenges in the future in Europe. Other work examining future orientated work on AMR has identified similar strategic considerations including – dedicated action to achieve positive outcomes from national and international organisations (e.g., EU) (12), global collaboration and a One Health approach (9), optimisation of prescribing habits and social norms (46), ensure solidarity and cooperation between actors in a diverse industry, support the uptake and effective use of technology and data (21), and increased state intervention in animal health (47).

Overall, this suggests there is high degree of lock-in to a specific development trajectory with implications for the realm of plausible change and action. In part, this reflects the limited space for intervention created by dominant mode of conventional agriculture, the regulatory regime, production systems, value chain structures and market arrangements. Dynamics of transitional lock-in is a widely observed phenomenon in governance and transition studies. In the context of this study, it reflects a commitment to the existing model and direction of change, dominant structural conditions and consensus amongst key Norwegian stakeholders participating in the process about the desirable direction of travel. Equally, the process reveals that anxieties about the future Norwegian governance system are not linked to external shocks.

However, it raises two sets of questions. Firstly, the degree to which AMR governance in the future requires a fundamental reassessment of priorities, away from approaches that focus on securing the conventional production model in the face of animal health and AMR challenges, towards alternative production, regulatory and market arrangements. If the problem is maintaining capacity in the face of the negative effects resulting from existing trajectories of change within the Norwegian system, a problem facing the rest of Europe as well, then how sustainable and resilient are strategies predicated on not stopping that trajectory but rather trying to accommodate these changes. Secondly, the degree to which these strategies, if effectively implemented, would open alternative possibilities for both AMR and agricultural governance in Norway, and Europe. This might especially be the case where efforts to enhance solidarity, develop bottom-up adaptive capacity amongst farmers, that could create new possibilities for collective understanding and capacity that contest the top-down priorities that currently characterises European and Norwegian AMR governance.

Our research therefore highlights the importance of broader socio-economic and political conditions to the governance of AMR in livestock farming. Although improving biosecurity, rational prescribing and preventive veterinary health measures are key practices at the farm and veterinary level, widespread adoption is linked to the broader context. As farmers, veterinarians, industry stakeholders and public authorities in Europe continue to grapple with AMR and antibiotic use in livestock farming, it is important to attend to these broader structural conditions that facilitate or hinder adoption of key practices. This is in line with a growing body of social science literature that has positioned antibiotics as a form of infrastructure. Consequently, an emphasis on antibiotics as infrastructure has drawn attention to the ways in which antibiotics compensate for biological, social and economic vulnerabilities produced within diverse local and global food systems (48–51).

However, there has been less attention to the ways in which actors work to compensate for the reduction in antibiotics (52), in short, how antibiotics as infrastructure ‘in decline’ is rendered in practice. Our study highlights multiple forms of ‘compensation’, including through social networks, institutional, economic and regulatory arrangements and intangibles such as high trust between actors, in addition to practical measures, technologies and knowledge, that work to compensate for the restrictions and reductions in antibiotic use in livestock farming. This contrasts with a broader European Union focus on individual farm level measures, such as improved housing, biosecurity, animal health data and hygienic measures (5). An approach that relies on individual value chain actors adopting changes instead of engaging with systemic and social network solutions that are indicated through this study. Developing a systemic approach for compensating for reduced antibiotic use will likely become more urgent as the European Union moves closer to trying to achieve its Farm to Fork target of a further 50% antibiotic use reductions against 2018 levels. Reductions that require a further 5% reduction year-on-year (53). This study therefore suggests a need to examine the robustness and longevity of current AMR governance strategies against a systemic, evolving challenge.

Similarly planning for a just transition towards low antibiotic use dependency livestock farming systems globally requires understanding the contextually appropriate mechanisms, initiatives and social networks that can adequately compensate for the reduction in antibiotics and fairly distribute burdens, without jeopardising animal and human lives, farmer livelihoods and food security (13). Developments in AMR governance cannot follow a one-size-fits all approach, neither globally, nor even within national economies (50). Although applied in Norway, a high resource and institutional capacity setting, the core principles of participatory scenario planning are applicable to diverse settings, livestock sectors and actor coalitions. However, as the outcome of this research showed, there is a need to develop an approach that is transparent and sensitive to the influence of power dynamics, social and cultural norms in small group settings that might be relevant to a particular field and country context. Participatory scenario planning offers an approach through which to develop contextually appropriate governance strategies to address AMR, that fulfils the need to design approaches that are deliberative, responsive, collaborative and adaptive (54, 55), whilst potentially helping to identifying diverse ways to compensate fairly for reductions in antibiotic use in livestock farming.

## Data Availability

The raw data supporting the conclusions of this article will be made available by the authors, without undue reservation.

## Footnotes

^1^The Population Correction Unit (PCU) is a theoretical unit of measurement developed by the European Medicines Agency (EMA) in 2009 and adopted across Europe. This standardised unit allows data to be easily aggregated or compared.
