# Supplementary material for: Governing antimicrobial resistance in Norwegian livestock farming to 2050: a participatory strategy development approach
Source: Front Vet Sci. 2025 Aug 14;12:1616206. doi: 10.3389/fvets.2025.1616206 (PMC12390962; doi:10.3389/fvets.2025.1616206)
Supplement: Supplementary file 1 [file Data_Sheet_1.pdf]

Supplementary materials: Initial scenarios

The following material outlines the 4 initial scenarios as developed in the first workshop and then given added detail by the researchers in advance of the second workshop.

Summary of scenario axes:

- **Scenario 1- Holding course**: Moderate prioritisation of agriculture, Moderately protectionist, High trust
- **Scenario 2 – Losing ground**: Low prioritisation, Moderately protectionist, Low trust
- **Scenario 3 – Proactive governance**: High prioritisation , Moderately protectionist, High trust
- **Scenario 4- The Dream**: High prioritisation, Highly protectionist, High trust

Scenario 1 – Holding course (status quo)

Summary

Prioritisation of agriculture- (High vs Low policy support)	Mixed political response and short-term prioritisation of agriculture- reflecting a decline in the number of active farms and farmers, weakening links to agriculture and a resultant concentration of intensive agricultural activity in places where it does occur
Norway's response to global events (Open vs Protectionist	Moderate protectionism- uneven record in responding to global developments
Trust in government (High vs Low)	High trust in government, some tensions

The Future

In 2050 Norway finds itself manging several pressures caused by global developments and their national consequences. Norwegian government has had a uneven record in addressing emerging global and national challenges although Norway has avoided the more extreme social and economic consequences that have emerged. Although trust in the government remains high, there is significant tension in agricultural and rural communities that have continued to decline as Norway has urbanised, centralised and the farm economy remained periodically very challenging in contrast to other areas of the economy. Although Norway retains its highly protectionist regime in relation to agriculture, fiscal support has entered a cyclical trend in which periods of real term declines in farm incomes are periodically addressed through short term measures. Supermarkets have become key actors in the Norwegian food system at the expense of farmers and cooperatives. They have used their market power to contractually

integrate farmers into their own value chains, using these arrangements to set on-farm standards on a wide range of practices including animal health and antibiotic use.

Industry Structure

The number of active farms and farmers has declined, with waves of farmers exiting the industry during challenging periods. In some areas this has resulted in land abandonment and reforesting, in others a concentration of more intensive agricultural activity. The cycle of deteriorating economic conditions, often resulting in public protests by farmers, means there are periods in which short term measures seek to return agricultural incomes to the national average. However, with numbers of active farmers declining, and growing numbers of Norwegians having weakening links to agriculture, the political impact has been mixed in the context of broader societal tensions. This cycle has perpetuated the restructuring of Norwegian agriculture towards larger farms, with increased stocking numbers and density. Farmers are increasingly directly integrated into specific supermarket value chains through preferential contractual arrangements. This has further facilitated the centralisation of production. Farmers outside of these arrangements have struggled. However, these larger farms are reliant on renting significant additional land to meet their grazing and feed needs. Consequently, their financial position is often precarious and contingent on a large class of small rural landlords. The small and medium farms that do remain have had to diversify into niche food production, tourism, or other activities. Farming is primarily an additional part time activity as part of a broader range of activities to remain financially viable. This has been mirrored in veterinary coverage. Difficulty in training and recruiting livestock veterinarians has resulted in uneven vet coverage. Large parts of the country are served by mobile vets resulting in only periodic access to veterinary services which is expensive.

Resilience and Surveillance

The broader context remains challenging as multiple global and European crises continue to unfold increasing Norway's vulnerability to zoonotic and exotic disease, and economic pressures. Resourcing remains tight when it comes to animal health and AMR management and surveillance. While state veterinary capacities are strong, the ability to act across the country on animal health is increasingly undermined by the lack of private veterinarians in many rural areas. There are increasingly difficult decisions as to where to prioritise resource use between AMR surveillance, exotic and previously eradicated animal disease and preventative animal health programmes. Import of live animals remains low meaning there is a limited likelihood of importing exotic or other zoonotic disease through this route. Cross-border collaboration on animal health and AMR with Nordic nations remains strong.

Compliance and engagement with existing and new programmes and the capacity of farmers and farming organisations to implement measures is strained by a volatile food economy, loss of expertise as farmers have left the industry, and structural polarisation with good biosecurity and capacity on larger, more professionalised and capital-intensive operations, and more limited capacity on small scale, part-time and lower income farms. Voluntary eradication programmes have become all but impossible due to a lack of trust in the state by small farmers who feel abandoned, and the high financial risks the threat of large culls poses to the more intensive farms who rely instead on data intensive monitoring systems. However, increased stocking densities have raised concerns for animal welfare and disease resilience with the risk that outbreaks within intensive farms will result in larger numbers of animals being slaughter.

Opportunities

Capacity and competence among larger commercial farmers is high with regards to animal health and biosecurity.

Smaller number of large farms means that there is fewer data that is easier to trace to specific farms meaning potential for early AMR and animal disease detection and preventative measures.

Challenges

Industry is vulnerable to large disease outbreaks due to greater stocking densities and larger concentrated farms increasing the potential scale of disease spread.

The marginalisation of small farms means that action on AMR and antibiotic use is difficult for small farmers who increasingly struggle to comply with regulations and adopt technologies because of a lack of financial support.

Lower investment and state support has resulted in the loss of human resources, especially veterinary competence and infrastructure, as well as farmer expertise.

Large commercial farms are increasingly reliant on migrant labour to make up for a lack of Norwegians working in the sector.

Scenario 2 – Losing ground (pessimistic scenario)

Summary

Prioritisation of agriculture- (High vs Low policy support)	Low prioritisation - Managing agriculture is no longer a policy goal.
Norway's response to global events (Open vs Protectionist)	Moderately protectionist but Norway is struggling to respond to European and global crises.
Trust in government (High vs Low)	Low public trust in ability of state to act effectively due to lack of early action

The Future

In 2050 Norway finds itself increasingly struggling to respond to, and manage the consequences of, escalating European and global system crises[1] and their national effects. Successive Norwegian governments had proven slow to respond effectively to a 'new normal' of permanent multiple crises resulting in state failures in governing problems, such as high energy, fertiliser and feed costs, due to ineffectual policies based on old assumptions and a lack of early action in key policy areas. These shortcomings have seriously impacting farmers and the publics' trust in the ability of the state to act effectively. Equally, the requirement to import replacement animals introduced several endemic diseases, including BVD and scab, that had previously been eradicated. Although fiscal support for agriculture remains relatively generous by

[1] War, terrorism, climate change, extreme weather events, human and animal disease pandemics

international standards, due to both increased international obligations and demands on state resources in other parts of society, there has been a consistent trend of real term declines in financial support over the intervening 30 years. Maintaining agriculture throughout the land is no longer an agricultural policy goal. Agricultural economic conditions in Norway are very challenging for small and medium producers, and the livestock sectors are heavily reliant on price volatile imports of animal feed.

Industry Structure

The result has been significant polarisation in agriculture with two main types of farms dominating. Large scale commercial farms and small-scale farms comprising part time hobby farmers, niche food producers and subsistence smallholders. Overall, there has been a steep decline in the number of farmers and difficulty in attracting students to veterinary schools and agricultural colleges. Self-sufficiency in food has declined, in part due to changing diets which have created demand for products that cannot be produced in Norway. The low number of remaining large-scale commercial farms are geographically concentrated around centralised processing and veterinary infrastructure in the most productive areas. These farms are focused on producing animals with high productivity, high health and welfare status. Food processing and intensive commercial farms increasingly rely on foreign workers.

In contrast, small-scale farms are geographically dispersed and primarily rear small numbers of sheep, goats and outdoor pigs based on local breeding stock. Many peripheral areas have poor or no access to veterinary services and products. Black markets for antibiotics have emerged in response. Live animal imports remain very low, except for poultry farming which is reliant on imports of production stock through the breeding pyramid.

Resilience and Surveillance

Live animals imports remain low. There is a limited likelihood of importing exotic or other zoonotic disease through this route. However, several endemic diseases have been reintroduced to Norway. Compliance and engagement with new government initiatives and efforts to improve biosecurity and animal disease prevention have had uneven success owing to limited faith in state authorities due to past failures. The increased reliance on foreign workers in large scale intensive production systems creates new risks for incursions. Similarly, climate change impacts have begun to change the migrations of several wild species which could act as vectors of animla and AMR disease. Regulation of antibiotic use in agriculture remains strong in principle but a combination of the loss of trust in state authorities, increased financial pressures and endemic disease threats have increased use over time.

Although several conditions have worsened and the risks of new AMR types becoming established in Norway have increased, the likelihood of an AMR establishment or animal disease outbreak remains low due to the geographic, political and market conditions in Norway. However, the geographic concentration of large-scale commercial farms means that if an outbreak does happen then it can rapidly move through an area impacting large numbers of animals, with significant costs associated with disease control and subsequent restocking. A particularly large outbreak would be likely to permanently impair the ability of a sector to recover to previous levels. Farm economy is stressed by high costs of concentrated feed imports meaning investments in new infrastructure and measures is limited. The ability to apply an eradication and control programme to an AMR pathogen such as MRSA in pigs is likely to face difficulty and heavy resistance from industry. In contrast, when disease incursions happen on

small-holdings and hobby farms they often go unnoticed by wider authorities due to uneven veterinary and surveillance coverage.

Reduced resources means that state AMR surveillance have been de-prioritised with a loss of surveillance capacity and difficulty in attracting veterinary scientists to the area. Dairy, pig and poultry sectors have limited private AMR surveillance targeting select, high profile AMR pathogens within their supply chains. The result has been the weakening of national AMR surveillance capacity and its narrowing to become focused only on legally required AMR bacterial targets stipulated by the EU. While surveillance of antibiotic use coverage amongst large commercial farms is considered to be good, most small-scale farms fall are poorly covered by state or private surveillance and are considered to be of low priority. Resource limitations and a loss of veterinary capacity in certain areas means that data quality is uneven and unable to effectively identify problem farms.

Opportunities

Retail chains have the opportunity to strengthen their role in this context and set animal health, antibiotic use and AMR prioritise within their value chains.

Science and technology developments in diagnostics, vaccines and precision farming will advance creating opportunities for improved data collection, analysis and interpretation and preventative measures.

Experimentation with non-pharmaceutical disease control methods amongst smallholders.

Challenges

Disease detection and surveillance for non-notifiable diseases is neglected. Surveillance of AMR is limited to only a small number of the most concerning zoonotic pathogens.

Farmer and veterinary expertise is concentrated within the areas with the largest commercial farms with smaller farmers suffering in terms of their ability to access veterinary services and educational resources.

Limited resources for veterinary services means that passive surveillance submissions by private veterinarians have fallen.

Growing resistance to state led measures and initiatives is due to low trust amongst key stakeholders.

Scenario 3 – Proactive governance (optimistic scenario)

Summary

Prioritisation of agriculture-<br>(High vs Low policy support)	High priority given to food security and food safety and a technologically advanced and innovative livestock sector.
Norway's response to global events<br>(Open vs Protectionist)	Moderately protectionist: Norway is responding to European and global crises but takes a protectionist approach

Trust in government (High vs Low)	High trust in the state and good levels of engagement with public (and farming sector)

The Future

Norway in 2050 gives a high priority to food security and food safety. This is mirrored at the EU level. Agricultural policy remains protectionist, financial support of high and resources for animal disease and AMR prevention are high. Proactive action by state authorities and industry on AMR, animal disease, and high health and welfare production is important for justifying this support. This has facilitated the development of a technologically advanced and innovative livestock sectors. The application of biotechnology to the production of protein animal feed from non-food products has enabled the replacement of some previously imported feeds that are highly price volatile. Livestock production remains focused on national needs as export costs are prohibitive. However, the broader context remains challenging as multiple global and European crises continue to unfold increasing Norway's vulnerability to zoonotic and exotic disease, and economic pressures. Although oil production has declined in quantitative terms, volatility in the oil market means that the revenues generated have remained relatively favourable over time.

Industry Structure

Despite high levels of resource allocation and policy support the numbers of Norwegian farmers have continued a steady decline. Decline has been more pronounced amongst small and medium scale farms. Consolidation and centralisation have increased the numbers of animals and stocking density on remaining farms. However, countervailing pressure from consumers for more outdoor farming means that there has been an increase in outdoor rearing of pigs and chicken, and limited adoption of 360-day indoor systems for dairy and beef cattle. This has created different possibilities for the circulation of disease and increased feed costs per animal. Part-time small-scale farmers, particularly in the sheep and goat sector, continue to operate in small numbers, alongside small and medium scale niche food producers supply regional markets.

Prioritisation of national R&D investments has facilitated the development of several technologies suited to Norwegian conditions whilst good provision of agricultural loans means that capital investment in technology and modern infrastructure is good across all scales. Digitalisation has improved farmers overview of herd health and their ability to rapidly identify sickness but has increased costs of disease management due to the need for subscriptions to data handling services. Agriculture is still geographically dispersed with some centralisation caused by food processing infrastructure being rationalised and consolidated. Prioritisation by government means that farmers are well supported. Recruitment of veterinary students to large animal practices remains challenging. The state and industry maintain robust veterinary infrastructure and surveillance capacity. Farmers continue to have high trust in the state and have good levels of engagement and compliance with government and industry led initiatives and interventions to support rational antibiotic use, animal disease prevention and biosecurity. Live animal imports remain very low, except for poultry farming which is reliant on imports of production stock through the breeding pyramid.

Resilience and Surveillance

Import of live animals remains very low meaning there is a limited likelihood of importing exotic or other zoonotic disease through this route. However, climate change impacts have begun to change the migrations of several wild species which could act as vectors of AMR disease. Norwegian authorities continue to utilise eradication and control programmes to ensure the national herd is free of zoonotic and animal diseases when necessary. Regulation and oversight of antibiotic use in agriculture remains strong and use is low. However, there is reduced willingness to use resources for preventative animal disease programmes, biosecurity and breeding for high health in part due to complacency as endemic animal disease is low in most sectors and perceptions that such investments are unnecessary. As a result, despite good levels of engagement with government and industry programmes on AMR and animal disease, the adoption of new measures to prevent and limit risks is reduced to farmers who have had a recent acute issue and had to take reactive measures.

State and private veterinary capacities are good, with the state being responsible for AMR surveillance and its compliance with broader EU monitoring initiatives. Cross-boundary collaboration on animal health is high for example in relation to managing wild boar along the border with Sweden. This means that although the risks posed by AMR, exotic and endemic disease outbreaks have increased in 2050, Norway retains the capacity to rapidly identify and respond to emerging problems including in collaboration with its neighbours. AMR surveillance targets as established in the context of national and EU level priorities. Antibiotic use data is good and linked to specific farms.

Where disease outbreaks do occur, it is often very limited in geographic scope and scale of impact due to the dispersed farming landscape although increased stocking density and animal numbers has meant that the scale of impact has increased over time. Impacted livestock sectors are largely able to recover to previous levels due to good resourcing and government support of insurance to farmers.

Opportunities

Strong state and private veterinary capacities and diagnostic technology use mean that there is increased opportunity for early disease detection and reducing disease impact.

Data quality is good and there is significant potential to utilise this data to identify problem farms enabling targeted interventions.

High levels of trust means it is relatively straightforward to implement new measures and initiatives due to high levels of coordination and collaboration with stakeholders.

Challenges

Fiscal and regulatory support for agriculture places a considerable burden on state budgets and it is a challenge to maintain public and political support in the face of wider societal challenges that also demand public resources.

Persistent concern for animal welfare has shifted focus away from animal health.

Norwegians, who have remained relatively affluent continue to have higher relative levels of international travel.

There are challenges maintaining national disease free status in the face of growing endemic and exotic disease threats.

Recruiting veterinarians to large animal production means there is a small pool of veterinarians involved in practice work.

Scenario 4 – Farmer renewal (dream scenario)

Summary

Prioritisation of agriculture-<br>(High vs Low policy support)	High priority given to food security and food safety as a key component of national security
Norway's response to global events<br>(Open vs Protectionist)	High levels of protectionism and prioritisation of self-sufficiency measures (reduced reliance on imports)
Trust in government<br>(High vs Low)	High trust in the state and good levels of engagement and collaboration with public (and farming sector), science and policy stakeholders

The Future

In response to several global systemic crises which had demonstrated European and Norwegian vulnerability in key areas Norway has highly prioritised food security as a key component of national security. Instability in the global marketplace caused by climate change, and conflict in Europe has resulted in high levels of protectionism and prioritisation of self-sufficiency measures. In 2050 this policy agenda has been aided by the Norwegian climate becoming more favourable for agricultural production, whereas biotechnological conversion of seaweeds has created new sources of feed. Collectively this has reduced reliance on imported animal feeds. Collaboration between the state and agricultural industry actors has continued to adopt an anticipatory and preventative policy approach in several areas, including animal disease and AMR. Norway retains sovereignty over agriculture and trade policy and has diverged in key areas with the EU where agricultural and food policy was deemed to threaten Norwegian production or incompatible with Norwegian conditions. Antibiotic use remains highly regulated because it has lots of public and industry support. Projecting a picture of high animal health and welfare and proactive action on AMR is a core part of differentiating Norwegian agriculture and justifying high levels of support. Although the broader global and European context remains challenging Norway has been a beneficiary of high and volatile oil and energy prices and thus government revenues remain favourable.

Industry Structure

With self-sufficiency concerns being high on the political agenda, and due to its limited agricultural resources, there has been a concerted effort to ensure that agriculture is maintained throughout the country to maximise resource use. High levels of direct financial support, high levels of promotion of Norwegian food, regional food strategies connecting

smaller food producers with local consumers alongside the maintenance of import trade tariffs have largely stabilise farm numbers whilst succession policies and support for new entrants has resulted in farmer demographics shifting slightly younger. The supported agricultural economy has enabled small and medium size farms to continue, as well as the maintenance of a distributed food processing infrastructure. Consequently, livestock production is still geographically dispersed although there has been some centralisation in the most productive areas to maximise resource efficiency. Prioritisation of national R&D investments has facilitated the development of some technologies suited to Norwegian conditions, but the relatively small market for pharmaceuticals in Norway means that access to agricultural antibiotics and other medicines is difficult and increasingly expensive. Good provision of agricultural loans means that capital investment in diagnostic technology and modern infrastructure is good across the sectors. Live animal imports remain very low, except for poultry farming which remains reliant on imports of production stock.

Resilience and Surveillance

Norway to maintains surveillance capacity over a wider array of AMR targets than those that are deemed domestically importance. However, there are considerable concerns about exotic disease. The result is significant investment in control measures and cross border collaboration to ensure that Norway remains free of several emerging and re-emerging pathogens. Particular attention is given to the spread of African Swine fever with routine culls of wild boar in Norway and considerable collaboration with Sweden to reduce likelihood of cross border migration. Although domestic antibiotic use and AMR remain important elements of surveillance the emphasis has shifted conclusively towards transmission into Norway. The state has therefore undertaken more routine surveillance of wildlife, the environment, and imported livestock.

Eradication and control programmes are in place for several diseases (inc. AMR), state surveillance infrastructure is strong and well resourced, and education on disease prevention, rational antibiotic use and biosecurity are routine. This is aided by both investments to facilitate technologically assisted farming which means on farm and national surveillance data is of high quality, and good farm economy which enables farmers to have the time and income to make necessary investments and do the work of ensuring good animal health and welfare. Although the state is responsible for exotic and AMR surveillance and compensation for eradication, industry continues to be responsible for several endemic disease surveillance and education programmes.

Although likelihood of AMR, exotic and endemic disease outbreaks are low, if they do occur their spread is limited by the geographic distribution of agriculture on the one hand and the ability of on farm and broader animal disease surveillance to rapidly identify events of concern. However, the gradual deterioration of the environment and climate change impacts means that there are increased concerns around sheep, goat and reindeer farming due to extensive rearing bring these animals into greater contact with wildlife, including migratory species. Sporadic endemic disease outbreaks continue to be a problem for chicken, pig and dairy production with antibiotic use increasing periodically in response. The industry is usually able to make a rapid recovery from such events due to their regional limitations allowing rapid restocking from other parts of Norway. However, due to difficulties in sourcing antibiotics that are tailored to the Norwegian policy and practical context medicine use is becoming increasingly expensive option for farmers to take to treat animals. This has redefined the threshold at which animals are sent to slaughter where previously they would continue to receive treatment, increasing routine losses.

Opportunities

Robust farm economy means that farmers can invest in and adopt to new technologies, diagnostics, and regulations.

New types of training initiatives related to precision farming technologies for the collection, analysis, and interpretation of animal health data.

Despite growing external risks, the industry is resilient in the face of disease outbreak disruptions and endemic disease is low.

High levels of trust and collaboration between science-policy-industry on key disease and AMR related issues.

High prioritisation of AMR surveillance means that there are good opportunities to discover AMR bacteria and intervene early to address them.

Challenges

The industry is vulnerable to government resource de-prioritisation. Fiscal and regulatory support for agriculture places a considerable burden on state budgets and it is a challenge to maintain public and political support in the face of wider societal challenges that are also demanding on public resources.

Norway could find itself increasingly internationally isolated in relation to its agricultural and food policies and under international pressure to reduce its agricultural protections in alignment with WTO and EU frameworks.
